# Exploration of Cold Extrusion for the Preparation of Enteric Minitablets of Isoniazid

**DOI:** 10.4103/0250-474X.42975

**Published:** 2008

**Authors:** M. C. Gohel, K. G. Sarvaiya

**Affiliations:** Department of Pharmaceutics, L. M. College of Pharmacy, P.O. Box. No. 4011, Navrangpura, Ahmedabad-380009, India

**Keywords:** Isoniazid, cold extrusion, minitablets, enteric, rifampicin

## Abstract

The objective of the present work was to formulate the enteric minitablets of isoniazid by cold extrusion method. The minitablets were prepared using isoniazid, hydroxylpropylmethylcellulose phthalate and dibasic calcium phosphate. The minitablets were coated using hydroxypropylmethylcellulose phthalate. Full factorial design was adopted to optimize the formulation. The minitablets showed good flow and acceptable friability. The drug release was resisted in 0.1 N HCl for 2 h from the optimized batch. The optimized batch showed more than 90% of drug release in phosphate buffer in 15 min. Capsules containing rifampicin powder and enteric isoniazid minitablets showed complete drug release in acidic and alkaline media respectively. The process of cold extrusion appears to be an attractive alternative to by-pass the existing patents.

A particular challenge in pharmaceutical research is the development of site specific dosage forms that release active ingredients at the site of absorption (e.g. intestine or colon). The enteric dosage forms are usually developed to overcome problems such as gastric irritation, drug stability in gastro-intestinal tract, poor absorption or permeability and incompatibility with other drugs^1^. Enteric dosage forms are commercially available as tablet, capsule, pellet, microcapsules and microspheres. The most commonly used pH sensitive enteric polymers are cellulose acetate phthalate (CAP), cellulose acetate trimelliate (CAT), hydroxypropylmethylcellulose phthalate (HPMCP), and methacrylic acid copolymers. Enteric dosage forms can be prepared by using aqueous coating, organic solvent based coating, latex system and incorporation of enteric co-fillers^2^,^3^.

Shishoo and co-workers reported the incompatibility of rifampicin and isoniazid in acidic dissolution medium^4^. Singh and co-workers reported that rifampicin is well absorbed from the stomach due to its high solubility at pH 1-2. The investigators further reported that isoniazid is poorly absorbed from stomach, but it is well absorbed from all the three segments of intestine^5^. Isoniazid should be enteric coated to resolve the issue of poor bioavailability of rifampicin. The enteric minitablets of isoniazid were formulated using a cold extrusion method. The results of comparative evaluation of hard gelatin capsule containing enteric minitablets of isoniazid and rifampicin powder, hard gelatin capsule containing only rifampicin powder and hard gelatin capsule containing rifampicin and isoniazid powder are also included.

## MATERIALS AND METHODS

Isoniazid IP was gifted by Sunij Pharma Pvt. Ltd., Ahmedabad. Hydroxypropylmethylcellulose phthalate and dibasic calcium phosphate were received as gift from Cadila Health Care Ltd., Ahmedabad. Acetone, magnesium stearate and dibutyl phthalate were procured from S. D. Fine Chemicals Ltd., Boisar.

### Preparation of isoniazid minitablets:

Isoniazid, hydroxypropylmethylcellulose phthalate and dibasic calcium phosphate were mixed for 10 min. Distilled water (1 ml per g of isoniazid) was used to prepare wet mass for extrusion. The mass was extruded to yield 1 mm diameter rods. The rods were partially dried by storing them at ambient conditions for 10 min and then cut to the size of 1 mm using a sharp blade. The minitablets were dried in a microwave oven for 2 min at 90°.

### Evaluation of isoniazid minitablets:

The isoniazid minitablets were evaluated for friability, angle of repose, Carr’s index and Hausner ratio to check mechanical strength and flowability^6^–^8^. The friability of the tablets was determined using Roche friabilator (Electrolab, Mumbai). The angle of repose was determined using a funnel method. Carr’s index is 100 times the ratio of bulk density minus tapped density to bulk density. Hausner ratio is the ratio of bulk density to tapped density.

### Coating of isoniazid minitablets:

The enteric coating was deposited on the minitablets using coating solution containing 2% w/v of hydroxylpropylmethylcellulose phthalate in acetone. Dibutyl phthalate (40% by weight of polymer) was used as a plasticizer and magnesium stearate (10% by weight of polymer) was used to impart hydrophobicity and luster to the coat. The minitablets of isoniazid were coated by pan coating method. The composition of isoniazid minitablets is shown in Table 1. The isoniazid minitablets were filled in hard gelatin capsule by considering their theoretical weights as shown in Table 1.

**TABLE 1:** COMPOSITIONS OF ISONIAZID TABLETS

Batches	Isoniazid (mg)	DCP (mg)	HPMCP X_1_ (mg)	Weight gain* X_2_ (mg)	Time in h to release 90% drug (h)
1	300	75	150	33.75	0.1
2	300	75	150	67.50	0.35
3	300	75	150	101.25	0.65
4	300	75	225	33.75	1
5	300	75	225	67.50	1.25
6	300	75	225	101.25	1.6
7	300	75	300	33.75	1.85
8	300	75	300	67.50	2.25
9	300	75	300	101.25	2.5

DCP - Dibasic calcium phosphate, HPMCP - Hydroxypropylmethylcellulose phthalate,

* Weight gain after enteric coating

### *In vitro* drug release:

*In vitro* drug release study was carried out for 4 h. In the first two hours, the study was carried out in 900 ml of 0.1 N HCl (pH 1.5) and later the drug release study was carried out in 900 ml of phosphate buffer (pH 7.4) in USP dissolution apparatus XXIII (basket). The temperature of dissolution medium was maintained at 37±2° and agitation speed of the basket was 100 rpm. The time in h required to release 90% of drug was measured. Isoniazid was estimated by spectrophotometric method at λ_max_ 263 nm as per USP. Dual wavelength spectorphotometric method was adopted for the estimation of rifampicin^4^. The dissolution study was also carried out for hard gelatin capsule containing rifampicin powder and minitablets of isoniazid. The results of dissolution testing were compared with the dissolution data of capsule containing rifampicin powder and capsule containing rifampicin plus isoniazid powder (non-enteric).

## RESULTS AND DISCUSSION

The cold extrusion method is simple, convenient and continuous processing method for preparing minitablets. Due to extrusion of wet mass, the diameter of tablet can be easily maintained throughout processing. Remon and co-workers used cold extrusion method for the preparation of uncoated hydrochlorthiazide tablets^9^. Remon and co-workers concluded that cold extrusion method can be used as an alternative tablet production technique for ingredients with poor compaction properties^9^. A 3^2^ full factorial design was adopted to prepare nine batches of minitablets with different concentration of HPMCP in core (X_1_) and weight gain by minitablets due to enteric coating (X_2_). The time required to release 90% (t_90_) of isoniazid (Y) was selected as a dependent variable. Dibasic calcium phosphate was used as a high density compressible excipient (dipping agent). The minitablets floated on the dissolution medium (0.1 N HCl) when dibasic calcium phosphate was not used in the formulation. Enteric coating was essential since dibasic calcium phosphate is soluble in acidic medium. The purpose of incorporating enteric polymer in the core of the minitablets was to modulate the release of isoniazid in alkaline medium.

The angle of repose, Carr’s index and Hausner ratio for the minitablets of all the batches were found in the acceptable range (31-33°, 12-15 and 1.0-1.1 respectively). The flow of minitablets can be classified from good to excellent as per USP^6^. The friability of minitablets was 0.3-0.6%, which is in the acceptable range for tablets, i.e. less than 1%^10^. It is concluded that the minitablets of isoniazid exhibited sufficient mechanical strength to withstand impacts during coating process and capsule filling. The diameter of minitablets was kept less than 1 mm so that minitablets are emptied in intestine via pylorus opening^11^,^12^. Before depositing enteric coat on the minitablets, films were casted in glass Petri dishes for preliminary screening of excipients. The formulation of coating solution was optimized for plasticity of film. Dibutyl phthalate was tried at 30, 40 and 50% by weight of polymer (HPMCP). The film of the batch containing 40% by weight of polymer (HPMCP) showed non-brittle characteristics on folding. Hence, the optimized solution containing 2% w/v of HPMCP in acetone, 40% dibutyl phthalate (by weight of polymer), 20% titanium dioxide (by weight of polymer) and 10% magnesium stearate (by weight of polymer) was used to coat minitablets of isoniazid.

**Fig. 1:**
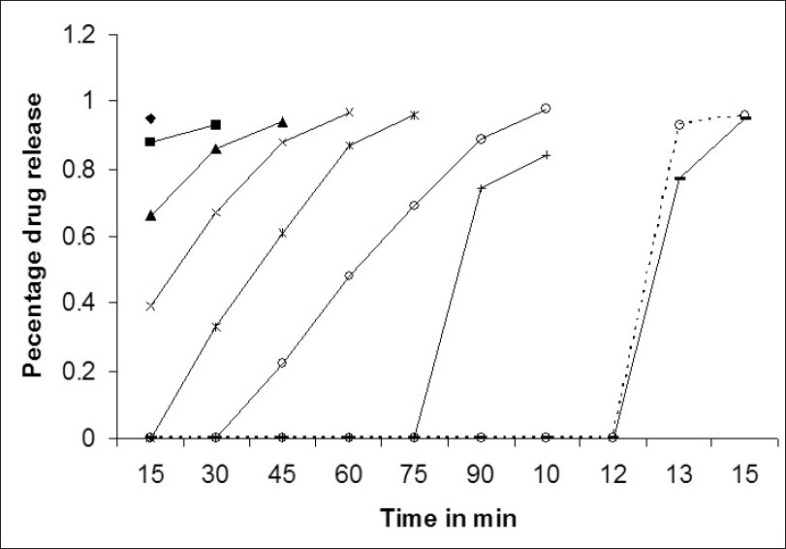
Dissolution profiles of different batches. 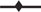
Batch 1, 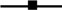
Batch 2, 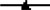
Batch 3, 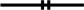
Batch 4, 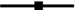
Batch 5, 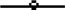
Batch 6, 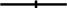
Batch 7, 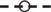
Batch 8 and 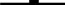
Batch 9

**Fig. 2:**
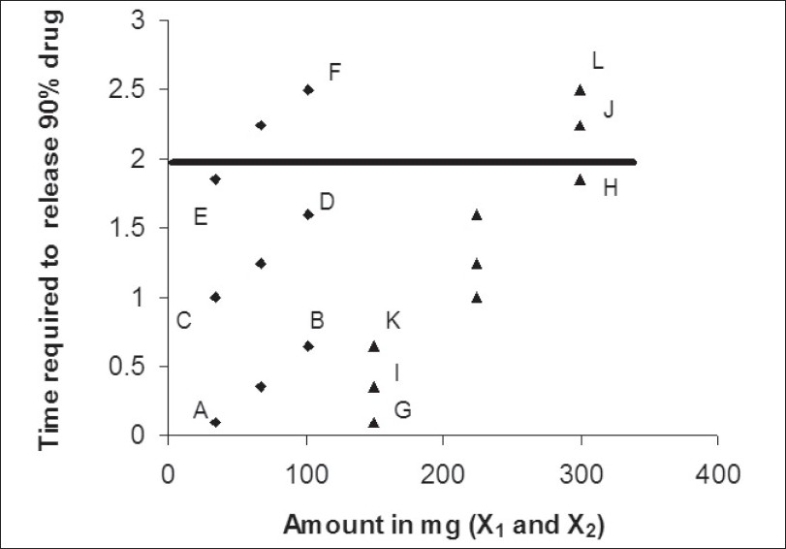
t_90_ versus variable X_1_ and X_2_. ▲ HPMCP concentration (X_1_) and ♦% Weight gain (X_2_)

**Fig. 3:**
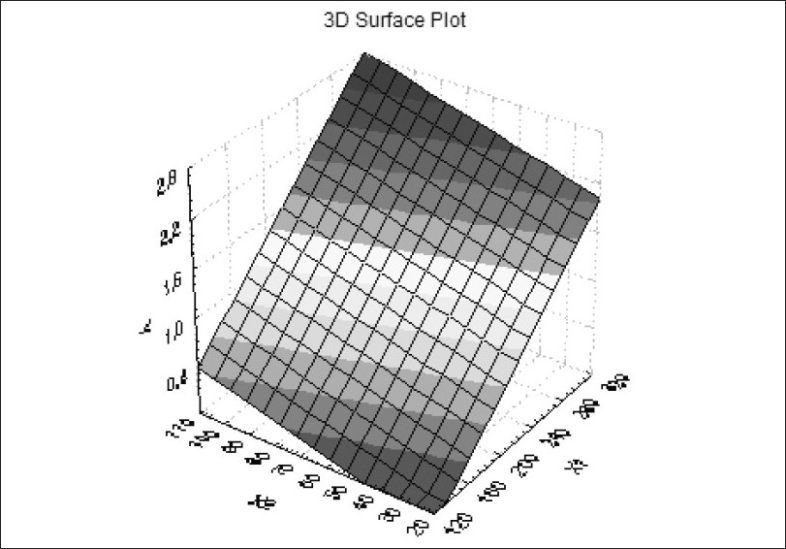
Surface plot for factorial design batches. 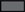
 0.073, 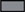 0.345, 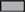 0.618, 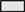 0.891, 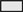 1.164, 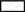 1.436, 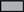 1.709, 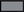 1.982, 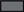 2.255, 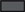 2.527 and 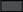 above.

**Fig. 4:**
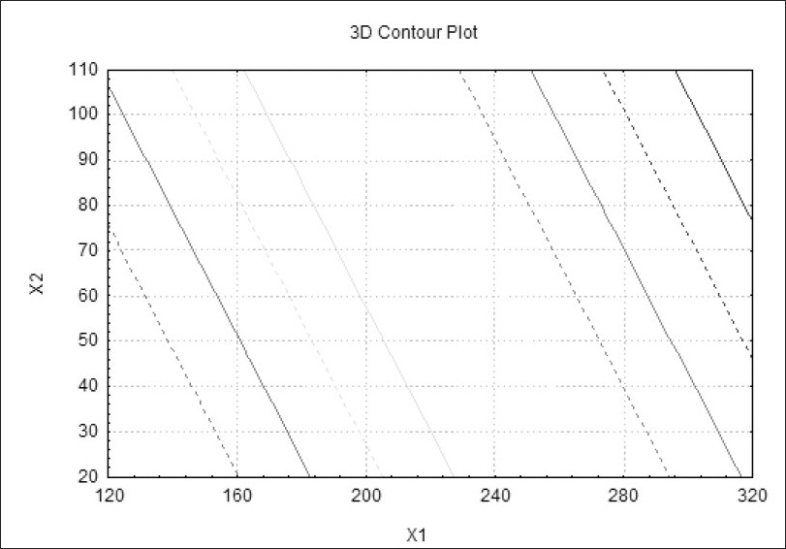
Contour plot for factorial design batches. The line shows t_90_ 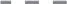 0.073, 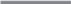 0.345, 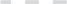 0.618, 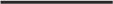 0.891,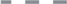 1.709, 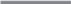 1.982, 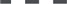 2.255 and 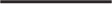 2.527.

Fig. 1 shows dissolution profile of the nine batches. The area above horizontal black line in fig. 2 is acceptance area for drug release from enteric minitablets of isoniazid. Greater than 90% isoniazid was released from the minitablets of batches 1 to 7 in the acidic dissolution medium in less than 2 h. The results (Table 1 and fig. 2) of batches 1-3, 4-6 and 7-9 reveal that t_90_ is dependent on weight gain by the minitablets. The results of batches 1, 4 and 7 reveal that t_90_ is also dependent on the amount of HPMCP in the minitablets. The batches 2, 5, and 8 and the batches 3, 6, and 9 showed similar trend. Out of bathes 8 and 9, batch 8 was selected for further studies since the t_90_ was less than that of batch 9 and it required less weight gain to modulate the drug release. The minitablets of batch 8 showed more than 90% drug release in phosphate buffer within 15 min and the drug release was resisted in 0.1 N HCl (pH 1.5) for 2 h (fig. 1). Therefore, batch 8 was ranked as best batch. The insolubility of HPMCP coat in acidic dissolution medium (0.1 N HCl) is responsible for retardation of drug release. It is reported that HPMCP has good acid resistance capacity and hence it is most widely used in enteric dosage form^13^. Figs. 3 and 4 shows the impact of percentage of HPMCP in core (X_1_) and weight gain (X_2_) on time required to release 90% of isoniazid (Y). Multiple regression analysis was performed to evolve mathematical model that correlates Y with X_1_ and X_2_. The regression coefficient (r) was 0.9992. The statistically insignificant terms such as X_1_ X_2_, X_1_^2^ and X_2_^2^ were eliminated from the full model because the P-values were greater than 0.05. The refined model is Y or (t_90_) = 0.012X_1_+0.009X_2_-2.067.

**Fig. 5:**
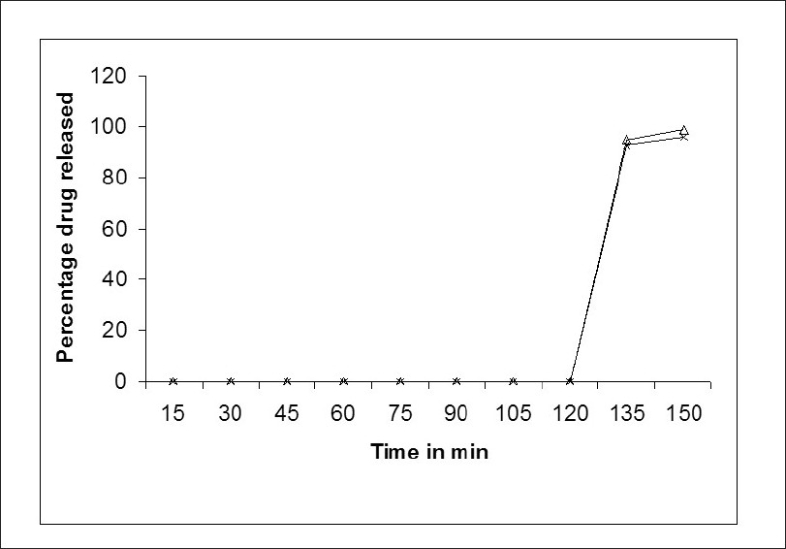
Comparison of dissolution profile of check-point batch and batch 8. 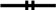
 Batch 8 and -△- Batch CP1

The results shown in Table 2 indicate that linear relationship exists between HPMCP concentration and weight gain. Hence, as the HPMCP concentration increases in core, less weight gain is required and vice-a-versa. One check-point batch (CP1) was prepared using 285 mg of HPMCP in core and 101mg weight gain. The observed and calculated values of Y were almost similar. The drug release profile of the check-point batch was comparable to that of batch 8 (fig. 5).

**TABLE 2:** REGRESSION ANALYSIS OF DATA POINTS SHOWN IN FIG. 2

Line	Equation	r^2^
AB	Y=0.0081X-0.1833	0.997
CD	Y=0.0089X-0.6833	0.990
EF	Y=0.0096X±1.55	0.982
GH	Y=0.0117X-1.6417	0.999
IJ	Y=0.0127X-1.5667	0.991
KL	Y=0.0123X-1.1917	0.999

r^2^ is the square of correlation coefficient.

**Fig. 6:**
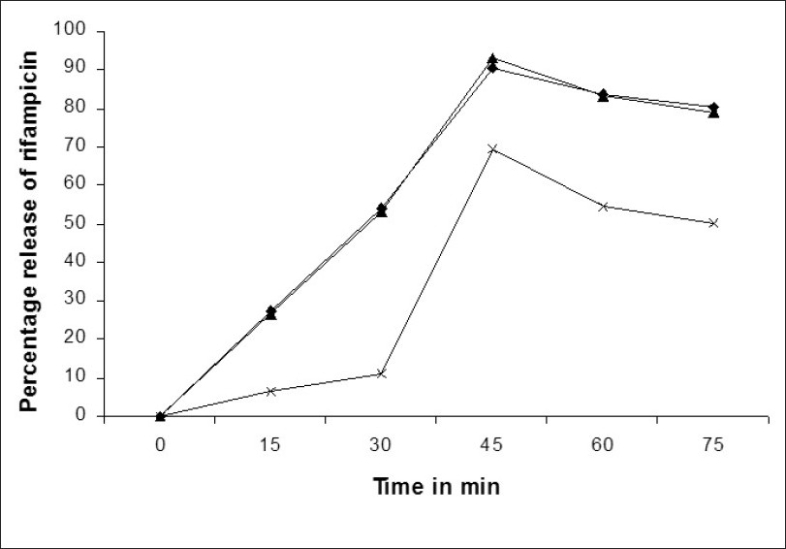
Comparative dissolution profiles of various formulations. 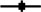
 Rifampicin in capsule, 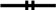 Rifampicin plus isoniazid capsule and 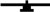
Rifampicin plus enteric isoniazid minitablets in capsule

Shishoo and co-workers reported that 12% rifampicin degraded to 3-formyl rifampicin sv (3FRSV) in acidic medium in 45 min while 21% of rifampicin was degraded in 45 min when rifampicin release study was carried out in presence of isoniazid^4^. Singh and co-workers reported that 17-24% of rifampicin degraded in 0.1 N HCl at 37° in 50 min when rifampicin was formulated with isoniazid^14^. Fig. 6 shows that the dissolution profile of rifampicin capsule and the capsule containing rifampicin plus enteric minitablets of isoniazid was comparable (more than 90% of rifampicin release at 45 min) while rifampicin plus isoniazid capsule showed incomplete pure drug release (maximum 70% of rifampicin release was noted at 45 min). The probable reason for incomplete drug release is the degradation of rifampicin to 3 FRSV. Isoniazid was not released in acidic dissolution medium from the enteric minitablets of isoniazid (batch 8) in 2 h. Therefore, the degradation of rifampicin due to interaction with isonicotinyl hydrazones (converted product of isoniazid in acidic condition) is arrested. The mechanism of degradation of rifampicin in presence of isoniazid has been reported by Singh *et al*^15^. Enteric coating of isoniazid is justified because the present study underlines the fact that minimization of contact between rifampicin and isoniazid in acidic condition results in less degradation of rifampicin.

The enteric quality was strongly influenced by the amount of hydoxypropylmethylcellulose phthalate in core and coat. The results of multiple regression analysis reveal that linear relationship exist between the amount of HPMCP in core and coat and the time required for 90% drug release. The degradation of rifampicin can be retarded to some extent in fixed dose combination formulations containing rifampicin and enteric coated isoniazid.
